# Developing sustainable Emergency Urology Simulation Training in sub‐Saharan Africa

**DOI:** 10.1002/bco2.70189

**Published:** 2026-03-10

**Authors:** William James Gladstone Finch, Tilaneh Leyeh Demilow, Ramzi Yesuf, Chales Mabedi, Linda Kayange, Vincent Medeyi, Getaneh Tesfaye Teferi, Fitsum Gebreegziabher Gebrehiwot, Folk‐Man Wong, Matthew Trail, Stephen R. Payne, Chandra Shekhar Biyani

**Affiliations:** ^1^ Norfolk and Norwich University Hospital Norwich UK; ^2^ Hawassa University Comprehensive Specialized Hospital Hawassa Ethiopia; ^3^ St. Paul's Hospital Millennium Medical College Addis Ababa Ethiopia; ^4^ Kamuzu Central Hospital Lilongwe Malawi; ^5^ Mengo Hospital Kampala Uganda; ^6^ MetaGlobe Academy Inc. New York City New York USA; ^7^ Western General Hospital Edinburgh UK; ^8^ Manchester University NHS Foundation Trust Manchester UK; ^9^ Urolink British Association of Urological Surgeons London UK; ^10^ St James's University Hospital Leeds UK; ^11^ Co‐Director CADSIM (Advanced Cadaveric Surgical Simulation Program), HEE Yorkshire & Humber, Level 9, Anatomy Department University of Leeds Leeds UK

**Keywords:** emergency, global, simulation, sustainability, urology

## Abstract

**Background:**

Simulation‐based education (SBE) is widely adopted in high‐income countries to enhance surgical training, but opportunities remain limited in low‐ and middle‐income countries (LMICs). Emergency Urology Simulation Training (EUST) was developed to address knowledge and skill gaps in managing urological emergencies in sub‐Saharan Africa.

**Methods:**

EUST courses were delivered in Ethiopia, Uganda and Malawi using a single‐day format combining pre‐course online learning, didactic teaching with hands‐on simulation. Locally, sourced animal tissue models were employed to replicate ureteric, bladder, renal, scrotal and penile injury repairs. Pre‐ and post‐course assessments included multiple‐choice questions (MCQs) and confidence surveys. Post‐course composite scores were compared across sites using the Kruskal–Wallis test. Faculty feedback evaluated preparedness and sustainability.

**Results:**

A total of 46 participants completed EUST across four sites, baseline knowledge was low (mean pre‐course MCQ scores: 8.0–10.75/15). Post‐course scores improved (10.63–12.72/15), with knowledge gains of 15%–32%. Confidence in managing rare emergencies, such as penile fracture and ureteric reimplantation, increased universally; 100% of delegates recommended integrating EUST into national training curricula. Faculty anxiety about SBE delivery decreased post‐training, and senior trainees transitioned well into faculty roles, supporting ongoing sustainability. Cost‐effectiveness was achieved by using animal tissue and donated instruments, reducing reliance on expensive synthetic models.

**Conclusion:**

EUST is an affordable, scalable and effective model for surgical education in LMICs. The ‘See one, run one’ approach enables replication across both specialty and international boundaries and fosters sustainable local faculty development. EUST demonstrates a significant positive educational impact and offers a sustainable programme for improving emergency urology care in resource‐limited settings. Partnering with COSECSA facilitates a reduction in UK faculty requirement long‐term.

## 1 INTRODUCTION

Simulation‐based training is well‐established in high‐income countries (HICs) and associated surgical training programmes. The benefits for training, in shortening the learning curve, exposure to infrequently encountered clinical situations and improving clinical skills and patient care, are well documented.^1^, ^2^ Opportunities for simulation‐based education (SBE) in low and middle‐income countries (LMICs) have been limited until recently.^3^, ^4^ A new collaboration between The British Association of Urological Surgeons (BAUS) Urolink,^5^ the British Journal of Urology International (BJUI) and trusted LMIC partners in sub‐Saharan Africa has been developed to help teach skills in the management of urological emergencies. This report outlines experiences, outcomes and lessons learnt from a project focussed on the development and implementation of emergency urology simulation training using SBE in three countries: Ethiopia, Uganda and Malawi.

Urology Bootcamps, run in the United Kingdom, deliver technical and nontechnical simulation training in all aspects of subspecialist urology at the outset of UK specialist training, giving exposure to a simulated experience of a wide range of urological procedures and management scenarios.^6^ Specified urology training programmes do exist and are emerging across countries in sub‐Saharan Africa.^7^, ^8^ A needs assessment, however, has shown that there is a considerable appetite for specific education in the management of urological emergencies in sub‐Saharan Africa^9^ and a lot of day‐to‐day emergency urology continues to be provided by general surgeons, surgical specialty residents and clinical officers^10^ and not by specialist trained urologists.

Emergency Urological Simulation Training (EUST) evolved to provide systematised training in common urological emergencies for general surgical, urological and gynaecological trainees in LMICs, each of whom may encounter all or some of the issues felt to be important in the needs assessment^9^ (Table 1). Before setting up a course, there were extensive online discussions with partners in the LMIC, with formalisation of a timetable for course construction on the local site. Online meetings between United Kingdom and local teams needed to occur at least monthly to ensure that the expected timetabled course delivery targets were being achieved.

**TABLE 1:** The modular construction of the Emergency Urological Simulation Training following the areas felt to be important by a needs assessment^9^.

Educational modality	Module 1	Module 2
Didactic education	Management of ureteric injuries	Management of the acute scrotum
	Management of renal, bladder and urethral injuries	Management of penile fracture and priapism
SIM training	Uretero‐ureterostomy Uretero‐neocystostomy Boari flap construction	Orchiopexy for testicular torsion
	Renorrhaphy for renal laceration	Repair of penile fracture

This paper is centred on the authors' experiences during the project and research undertaken during that period, which examined the experiences of trainees and educators with simulation‐based teaching.

## 2 METHODS

### 2.1 Study design and setting

This was a prospective, multisite evaluation of EUST delivered at four African training locations: Hawassa (Ethiopia), Addis Ababa (Ethiopia), Kampala (Uganda) and Lilongwe (Malawi). Each site hosted a single course delivered using the same curriculum, learning objectives, educational materials and assessment framework. Participants attended only one course and did not cross between sites.

### 2.2 Participants

Participants were surgical trainees and early‐career surgeons involved in the management of urological emergencies. Participant composition varied by site, reflecting local training structures, specialty mix and educational priorities. Participants included urology trainees, general surgeons and gynaecologists, reflecting the range of clinicians responsible for managing emergency urological conditions in these settings. At some sites, selected participants were exposed to the programme with a view to future delivery or mentorship roles, consistent with a ‘see one, do one, mentor one’ approach. Participation in the evaluations was voluntary. Pre‐course and post‐course assessments were compared at each site, and no participant contributed data to more than one course. Pairing was verified locally using internal identifiers and confirmed analytically during data review.

### 2.3 Course contents

The training was designed to be provided in two modules (Table 1), deliverable in a single day, to minimise both the financial impact and time away from clinical activity for both learners and teachers.

The course was structured so that didactic education was provided using evidence‐based, standardised, PowerPoint slide sets in English. The SIM component was developed using medium fidelity fresh animal bench models^11^ simulating human anatomy.

### 2.4 Educational resourcing

**FIGURE 1:**
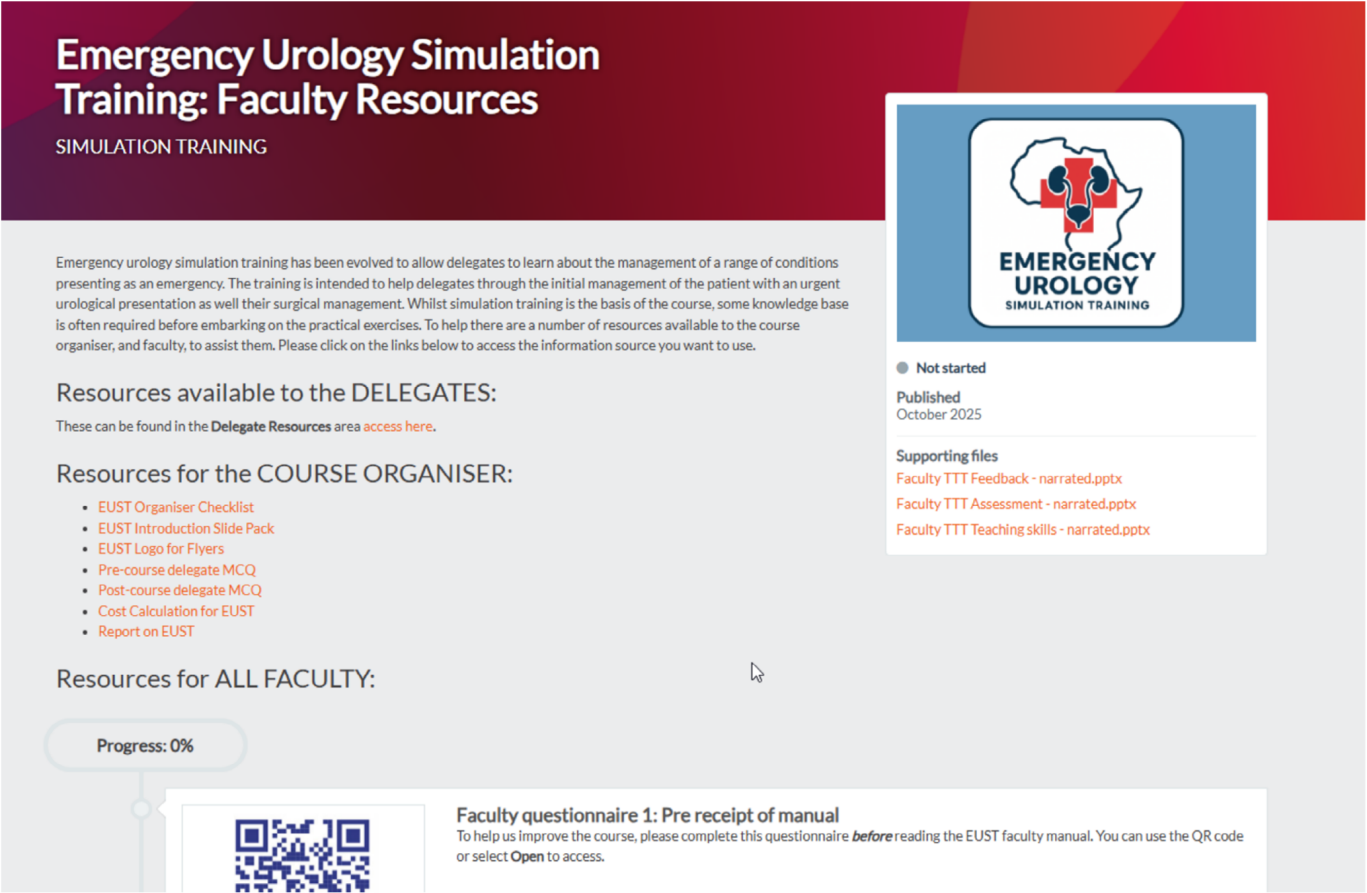
The online course website available to faculty. A ‘trimmed down’ version was available to delegates (Table 2).

Experience from the first two courses facilitated the development of an online resource ‘package’ that was thought to fulfil the needs of learners and educators receiving, or delivering, SIM education where resource infrastructure was poor. The partnership with BJUI facilitated the creation and development of a novel online portal (Figure 1), accessible to delegates and faculty after online course registration and provision of an email address. This portal acted as a resource repository for the course organiser, faculty and delegates (Table 2).

**TABLE 2:** Online educational resources available to the organiser, faculty and delegates taking part in the Emergency Urological Simulation Training (EUST).

Resource	Organiser	Faculty	Delegate
Course checklist	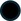		
Course introduction slide set	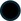		
Pre‐ and post‐course MCQs	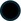	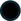	
Course manual	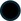	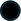	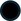
Module 1—Ureteric injury slide set	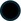	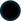	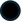
Module 1—Renal, bladder and urethral injury slide set	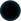	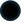	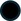
Module 2—The acute scrotum slide set	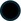	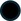	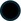
Module 2—Penile fracture and priapism slide set	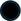	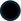	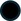

Abbreviation: MCQs, included multiple‐choice questions.

Delegates were able to access all the un‐narrated slide sets for reference before and after the course. The ability to access these sets meant that delegates could take full advantage of the faculty during a course without the need to take notes during interactive discussion. They also had access to a comprehensive course manual as part of their ‘pack’. This covered all common urological emergencies likely to be encountered. This included evaluation of the injured patient, basic surgical skills and catheterisation that were not specifically covered during the course.

Faculty had access to both un‐narrated and narrated versions of the slide sets so that delegates could still benefit from didactic teaching, should local faculty not be available to personally deliver the slide set during the course. The faculty version of the manual additionally contained details of course delivery and the set up for each simulation exercise, thereby acting as a blueprint for provision of an EUST.

### 2.5 Course delivery

Courses started with an introduction by the local organiser covering the educational validity of the course, its structure and its educational objectives, and it also clarified the educative needs of the learner cohort for that course.

**FIGURE 2:**
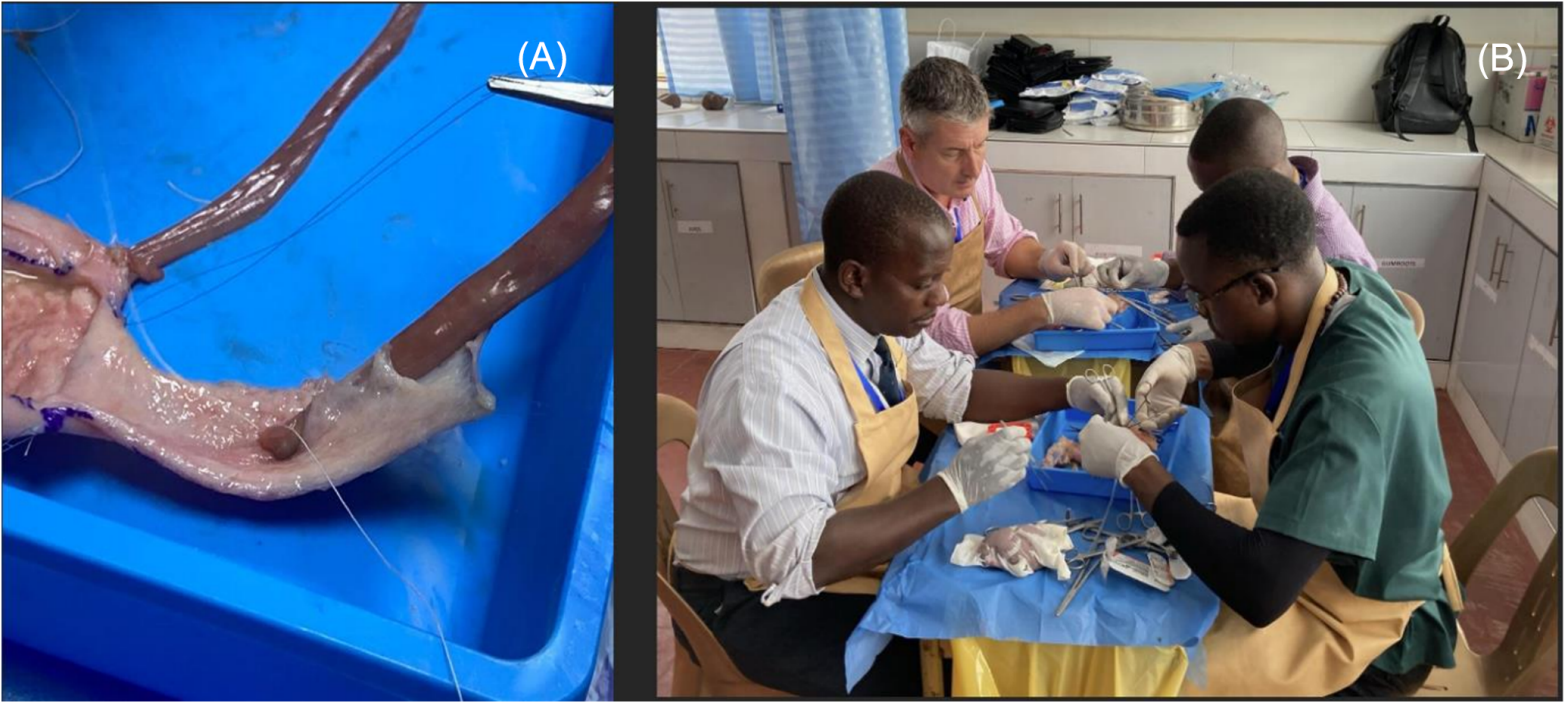
(A) Tunnelled uretero‐neocystostomy over a nasogastric tube into a Boari flap, utilising goat intestine as simulated ureter. (B) One‐on‐one faculty to delegate simulation training of how to perform an orchiopexy for torsion.

In Module 1, a lecture about the causes and investigation of ureteric injury was followed by simulated repair of both ureteric and bladder injuries utilising a goat bladder model, with goat intestine acting as a ureteric substitute. Ureteric anastomoses over a stent, ureteric reimplantation and Boari flap reconstruction were all performed in plastic trays (Figure 2A).

Delegates received 1:1 tuition with expert faculty, many of whom had been recruited locally and from surrounding hospitals to the course. Faculty included some senior trainees who were appropriately experienced to provide this form of education. Faculty provided invaluable knowledge, hands‐on skills and an opportunity for delegates to discuss, at length, topics related to the simulation tasks. A further short lecture about renal, bladder and urethral injury management was followed by repair of a simulated polar kidney laceration using a cow, pig or goat renal model.

In Module 2 a brief lecture on the management of the acute scrotum was followed by exploration of a goat, or bullock's, scrotum, teaching the principles of testicular fixation and testicular repair for traumatic rupture (Figure 2B). A talk on the management of penile fracture and priapism was followed by simulation of a corporal repair using a bull's penis as a model for fracture exposure, when the assessment and repair of concomitant urethral injury could be undertaken.

Delegates were divided into two groups, with a scheduled rotation taking place after lunch. Due to logistical considerations at the Uganda venue, the session structure was modified: All theoretical presentations were delivered at the start of the session. This was followed by a structured practical session, where all delegates first completed the ureteric injury practicals, followed by the Module 2 practical components.

The EUST one‐to‐one trainee‐to‐trainer model mirrored realistic clinical environments and is the preferred educative format if sufficient faculty are available. This enhances skill development by demonstrating essential, ‘deliberate’ practice and allows effective trainee feedback.^12^ This approach mitigated the effect of a heterogeneous trainee group, characterised by a variable level of prior experience, and is considerably better than having multiple trainees in a simulation session with one trainer. While that can decrease training time for a large cohort of trainees, it risks rendering sessions unproductive if trainees do not engage with the process due to their individual training needs not being met.

### 2.6 Determining educational value and course deliverability

The educational value of this approach for EUST was evaluated by both delegates and faculty as a routine quality analytic of course delivery and to help highlight areas of the course that require ongoing development.

Delegates were asked their individual expectations of the course at enrolment using a QR‐code driven Google Form link and the group's cumulative response was used to individualise course content. This questionnaire asked about their knowledge of and confidence in managing certain emergency urological conditions pre‐course. At course completion, this questionnaire was repeated, and delegates were also asked about their ability to translate the SIM skills they had accrued into everyday practice and their ability to teach colleagues what they had learnt. Responses were collected on a 5‐point Likert scale between (1) *Not at all confident* and (5) *Extremely confident*. Perceived knowledge and confidence were assessed using procedure‐specific Likert items. For each participant, composite scores for perceived knowledge and perceived confidence were calculated separately by taking the mean of all available procedure‐specific responses within each domain at each timepoint. All procedures were weighted equally. Where responses to individual procedures were missing, composite scores were calculated using the remaining available items.

To determine knowledge gain, delegates were asked to complete an online multiple‐choice question (MCQ) to act as a baseline indicator of their topic knowledge at the start of the course; it was repeated at the end of the SIM session. The MCQ consisted of identical single‐best‐answer questions across all four sites and both timepoints, with a maximum possible score of 15. MCQ data were analysed as paired observations within each site, and the knowledge gain was determined by the percentage change from baseline MCQ performance.

The faculty were asked to complete a QR‐code driven Google Form questionnaire about their experience of SIM training and their confidence in their ability to provide this form of education, both before and after the course. The questionnaire examined their clinical role, SIM training experience, understanding of what SIM training involved and their preparedness for a role as a SIM educator. They were also asked to evaluate their credibility and confidence in providing SIM training, their anxieties about technological malfunction, learner buy‐in, ability to provide appropriate feedback and institutional or personal reputational risks of underdelivery. Responses were collected on a 5‐point Likert scale between (1) *Strongly disagree* and (5) *Strongly agree*. Faculty were also requested to provide feedback about course organisation.

### 2.7 Data integrity and handling

All datasets underwent a structured integrity review prior to analysis to ensure that pre‐ and post‐course comparisons were valid and that outcome measures were interpreted consistently across sites. This process included verification that pre‐ and post‐course responses were correctly paired at the individual participant level, confirmation that MCQ scores fell within the valid range of the assessment instrument, and review of post‐course datasets to confirm completeness and correct site attribution. Differences in variable naming between datasets were reconciled to ensure consistent alignment of equivalent items across sites prior to analysis.

### 2.8 Statistical analysis

Given the small sample sizes at each site and the ordinal nature of Likert‐scale data, nonparametric methods were used for all inferential analyses. Pre‐ and post‐course MCQ scores were compared within each site using the Wilcoxon signed‐rank test to assess within‐participant change without assuming normal score distributions. Paired composite scores for perceived knowledge and confidence were analysed using the same approach. Post‐course composite scores were compared across sites using the Kruskal–Wallis test to explore consistency of outcomes across heterogeneous training environments, without assuming equivalence between cohorts.

For descriptive purposes, MCQ scores are reported as means with standard deviations to aid interpretation of absolute knowledge levels, while Likert composite scores are summarised using medians and interquartile ranges (IQRs). Statistical significance was defined as *p* < 0.05.

## 3 RESULTS

### 3.1 Participants and cohort composition

A total of 46 participants completed EUST across four sites, with cohort sizes ranging from 11 to 12 participants per location. Participant characteristics differed across sites with respect to training grade, specialty mix, gender balance and faculty delivery structure (Table 3).

**TABLE 3:** Participant composition, faculty delivery structure and baseline characteristics across Emergency Urological Simulation Training (EUST) sites (Knowledge gain (%) calculated as: (post‐course mean MCQ—pre‐course mean MCQ)/pre‐course mean MCQ × 100.).

	Hawassa (Ethiopia)	Addis Ababa (Ethiopia)	Uganda	Malawi
Participants (*n*)	11	12	12	11
Delegate profile	Residents/interns	Urology residents	Surgeons/residents	Urology/GS/OBGYN
Gender balance	Predominantly Male	Predominantly Male	85.7% Male	52.9% Female
Faculty ratio Local:Visiting faculty	8:5	7:4 (3 UK, 1 Uganda)	6:7 (6 UK, 1 Zimbabwe)	11:4
Pre‐course MCQ (Avg)	10.72/15	10.75/15	9.33/15	8.00/15
Post‐course MCQ (Avg)	12.72/15	12.41/15	11.33/15	10.63/15
Knowledge gain (%)	18.65%	15.46%	21.43%	32.87%
Objectives met (%)	100%	95.2%	78.6% (Strongly Agree)	95.3%

The Hawassa and Addis Ababa cohorts consisted predominantly of urology trainees. In contrast, the Uganda and Malawi cohorts included more heterogeneous groups spanning urology, general surgery and obstetrics and gynaecology, reflecting the range of clinicians involved in delivering emergency urological care in these settings. Local‐to‐visiting faculty ratios also varied across sites, indicating differences in local capacity and stage of programme implementation.

### 3.2 Objective knowledge outcomes (MCQ)

Paired pre‐ and post‐course MCQ data were available for all four sites. At each location, mean MCQ scores were higher after EUST training than before (Table 4). On paired analysis, these improvements were statistically significant at all sites.

**TABLE 4:** Pre‐ and post‐course multiple‐choice question (MCQ) scores by site.

Site	*n*	Pre‐course mean (SD)	Post‐course mean (SD)	Mean change (95% CI)	*p*
Malawi	11	8.1	10.7	+2.6 (1.0–4.3)	<0.01
Uganda	12	9.2	11.2	+2.0 (1.2–2.8)	<0.01
Addis Ababa	12	10.3	12.0	+1.7 (0.7–2.6)	<0.01
Hawassa	11	10.6	12.6	+2.0 (1.1–2.9)	<0.01

**FIGURE 3:**
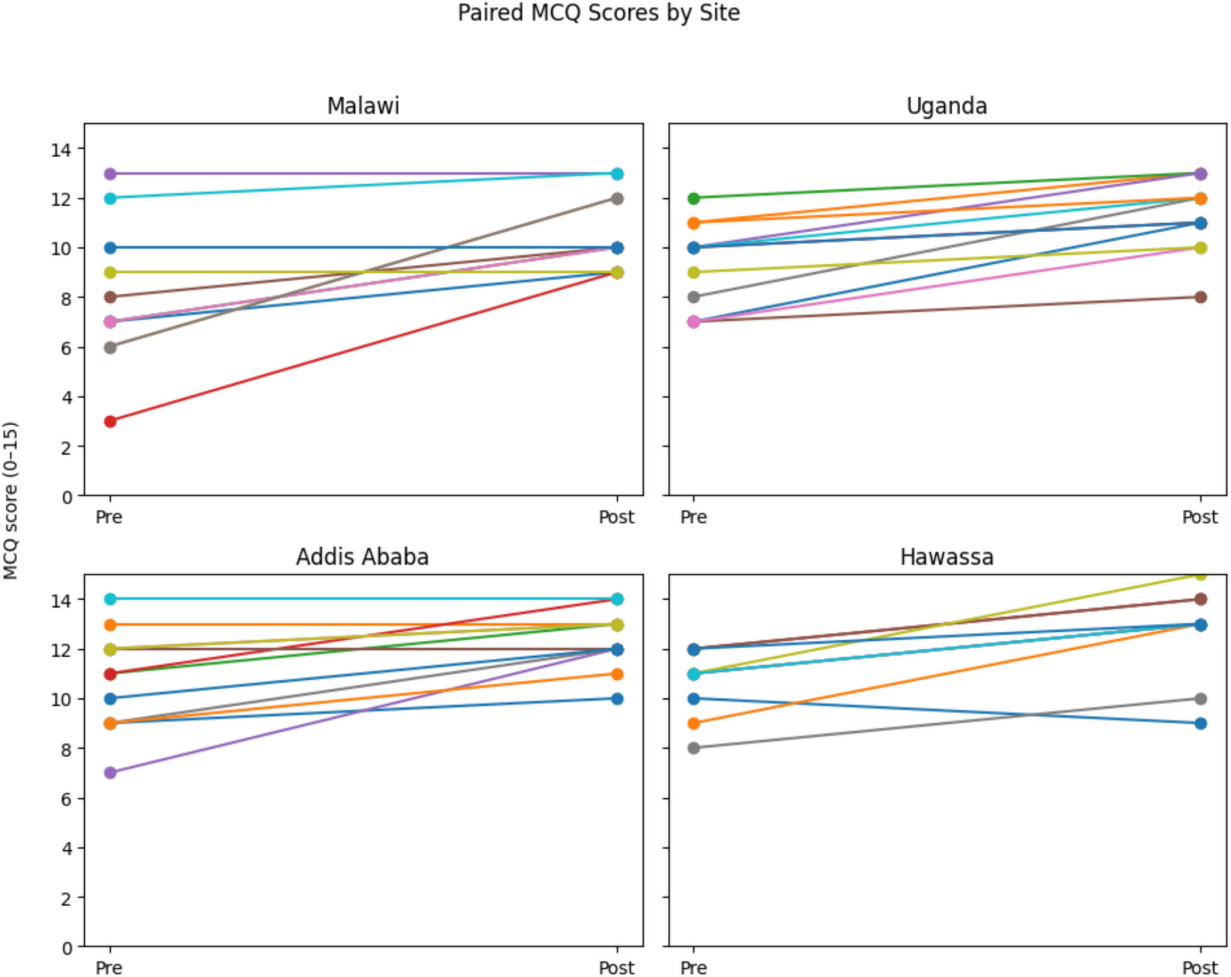
Individual pre‐ and post‐course MCQ scores by site. Paired line plots showing participant MCQ scores before and after Emergency Urological Simulation Training (EUST) training at each site (maximum score = 15). Each line represents one participant.

When individual participant scores were examined, most participants showed improvement from pre‐ to post‐course assessment (Figure 3). This pattern was observed consistently across all sites, despite differences in baseline MCQ performance and participant composition.

MCQ scores increased significantly after training at all sites, even though starting knowledge levels differed between cohorts.

### 3.3 Subjective preparedness outcomes

#### 3.3.1 Perceived knowledge

Paired pre‐ and post‐course composite scores for perceived knowledge were available for all sites (Table 5).

**TABLE 5:** Paired pre‐ and post‐course composite Likert scores for perceived knowledge and confidence. Composite scores were calculated as the mean of available procedure‐specific Likert responses per participant at each timepoint. Higher scores indicate greater perceived knowledge or confidence. Wilcoxon signed‐rank tests compare paired pre‐ and post‐course scores within sites.

Site	Outcome	Paired n	Pre‐course median (IQR)	Post‐course median (IQR)	Wilcoxon *p*
**Malawi (Kamuzu)**	Perceived knowledge	11	2.50 (2.12–3.06)	4.12 (3.75–4.88)	0.0186
	Confidence	11	1.88 (1.62–2.38)	4.62 (3.94–4.94)	0.0049
**Uganda (Mengo)**	Perceived knowledge	11	2.88 (2.12–3.75)	4.62 (4.25–4.69)	0.0068
	Confidence	11	2.88 (1.88–3.62)	4.50 (4.25–5.00)	0.0051
**Addis Ababa (Ethiopia)**	Perceived knowledge	12	3.69 (3.12–4.16)	4.88 (4.53–5.00)	0.0122
	Confidence	12	3.69 (2.19–4.03)	4.81 (4.50–4.91)	0.0066
**Hawassa (Ethiopia)**	Perceived knowledge	10	4.44 (3.91–4.72)	4.44 (3.81–4.69)	0.6733
	Confidence	10	4.25 (3.81–4.72)	4.25 (3.75–4.72)	0.9325

**FIGURE 4:**
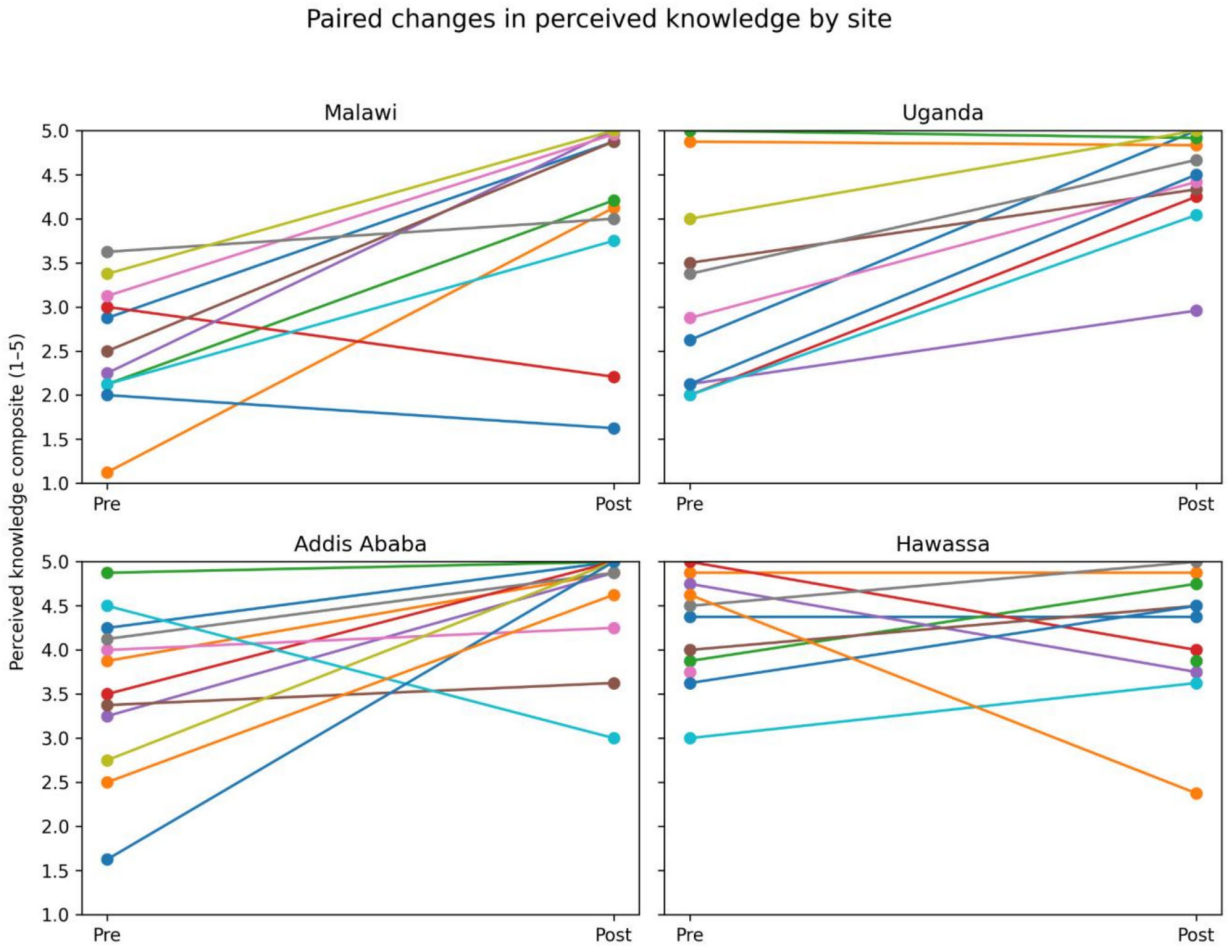
Paired changes in perceived knowledge composite scores before and after Emergency Urological Simulation Training (EUST) training. Each line represents one participant. Composite scores are calculated as the mean of procedure‐specific Likert responses.

Participants who reported lower levels of perceived knowledge before the course showed increases following training, whereas participants with higher baseline self‐ratings remained at similarly high levels after the course (Figure 4).

In Hawassa, baseline perceived knowledge scores were high prior to training and remained high at the post‐course assessment. No statistically significant change was detected for perceived knowledge at this site.

#### 3.3.2 Confidence

**FIGURE 5:**
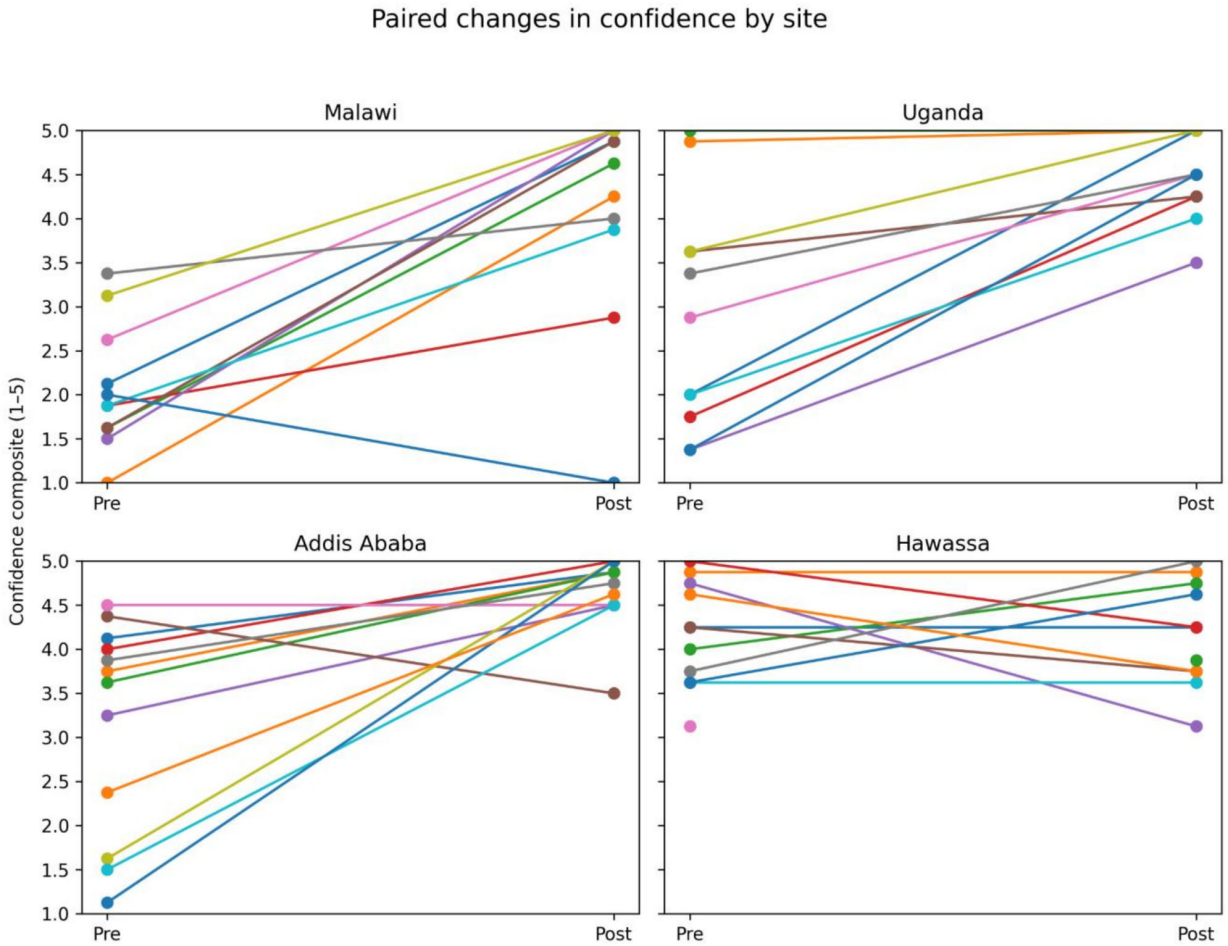
Paired changes in confidence composite scores before and after Emergency Urological Simulation Training (EUST) training. Each line represents one participant. Composite scores are calculated as the mean of procedure‐specific Likert responses. One Hawassa participant did not provide complete paired Likert data, resulting in a paired sample of *n* = 10 for subjective outcomes at that site.

A similar pattern was observed for confidence outcomes (Table 5). Participants who initially reported lower confidence demonstrated increases following training, while those with higher baseline confidence showed little change and remained at high post‐course levels (Figure 5). In Hawassa, confidence scores were high at baseline and did not change significantly following training, consistent with the observed pattern for perceived knowledge at this site. One participant in the Hawassa cohort did not provide complete paired Likert data, resulting in a paired sample size of *n* = 10 for subjective outcomes at that site.

**FIGURE 6:**
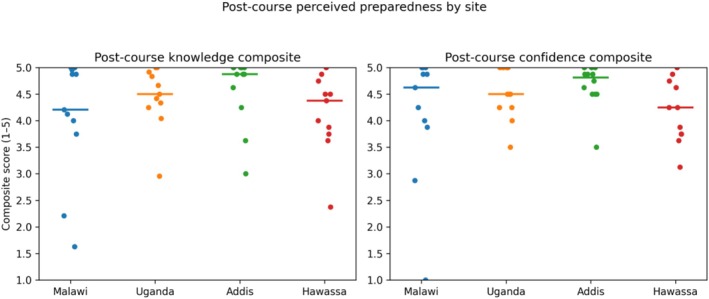
Post‐course perceived knowledge composite scores by site. Note: These findings should be interpreted in the context of small site‐level sample sizes and variation in participant composition.

Paired pre‐ and post‐course composite Likert scores for perceived knowledge and confidence demonstrated statistically significant improvements in Malawi, Uganda and Addis Ababa. No statistically significant pre–post change was detected in Hawassa for either domain. Post‐course perceived knowledge composite scores were high across all four sites (Figure 6). When post‐course scores were compared across locations, no statistically detectable differences were observed (using Kruskal–Wallis).

Dot plot showing the distribution of individual post‐course perceived knowledge composite scores across EUST sites. Horizontal lines indicate site‐specific medians.

Integration of objective and subjective outcomes across all sites, objective MCQ scores improved following EUST training, including in cohorts where baseline self‐reported preparedness was already high. In Addis Ababa and Hawassa, objective knowledge gains were observed despite limited detectable change in perceived knowledge and confidence scores. This pattern is consistent with ceiling effects and/or limited sensitivity of self‐report measures in higher prepared cohorts, rather than absence of educational impact.

### 3.4 Faculty confidence

12.5% of 24 faculty had no SBE experience prior to the courses, 66.7% had some, 4.2% a moderate amount and 16.7% a fair amount of familiarity with this as an educational methodology. Prior to the course, faculty had some concerns about providing SBE, predominantly related to their familiarity with SBE as a teaching modality, their ability to provide teaching utilising this educative format, its costs and its technical complexity.

Post‐course questionnaires demonstrated continued low levels of anxiety about providing education utilising simulation models. The trained surgeon component of faculty had marginally higher anxiety about providing the course (mean Likert score 1.71 prior to the course and 1.95 afterwards), whereas trainee faculty demonstrated a reduction in anxiety (mean Likert score 1.65 prior to the course and 1.38 afterwards). Both groups wanted additional resources to be able to provide SBE; many had been unable to access the parallel online material that had been provided, for technical reasons predominantly related to internet accessibility and download speeds. There was a considerable hunger for greater knowledge about educational theory via a ‘Training the Trainers’ type of course to help support the simulation training.

Global faculty feedback was, however, almost universally positive, with many expressing this was a highly relevant way of providing surgical education, and a methodology they were very keen to perpetuate.

### 3.5 Course costing

All simulation teaching materials were available locally. The local cost for running the course varied from £500 ($669) to £2000 ($2676) excluding the travelling cost of the UK faculty mentoring each course at a new site.

## 4 DISCUSSION

### 4.1 Models of course dissemination

The 1st EUST in a LMIC was held in Hawassa, Ethiopia in November 2024, where Urolink had a relationship for >15 years.^5^ After considerable online discussion, the course was developed and organised according to the desired outcome from the needs assessment. The local team of three urologists was mentored by four UK urologists: two colleagues from Addis Ababa acted as invited faculty with the intention of providing a subsequent course in their locality.

The Addis team went on to host and run the 2nd EUST in May 2025, proving that the course could be run identically in the same country, with the same level of benefit to learners and without a long‐term Urolink relationship. During that second course, a urologist from Kampala, Uganda, attended as invited faculty.

This ‘See one’ approach enabled our Ugandan colleague to run the 3rd EUST at Mengo Hospital, Kampala in November 2025 with fewer UK mentors directly contributing to the course. This proved that the EUST model could be replicated across international borders.

A 4th EUST course was successfully run at Kamuzu Central Hospital in Lilongwe Malawi, also in November 2025. Like the team in Hawassa, Lilongwe has a well‐established Urolink relationship and was able to demonstrate that the programme could be delivered without having seen a EUST, as long as remote mentoring and an online portal were available for the first course.

There is a plan to deliver two courses in Ethiopia (Hawassa and Addis Ababa) in early 2026 and will be mentored and run by local faculty with an observational, light touch role planned for a limited UK faculty. The hope is that this will demonstrate the successful transition of knowledge and delivery of EUST using a ‘see one, run one, mentor one’ approach.

This multi‐site evaluation demonstrates that EUST can be delivered across diverse African training environments with reproducible educational gains. Objective knowledge improvement was observed consistently across all four sites using an identical assessment instrument, despite differences in baseline preparedness, participant composition and faculty delivery structure.

Improvements in perceived knowledge and confidence were greatest in cohorts with lower baseline self‐ratings, while ceiling effects limited the sensitivity of subjective measures in higher prepared groups. Importantly, objective knowledge gains were still observed in these cohorts, highlighting the complementary roles of objective and self‐reported outcome measures when evaluating educational interventions.

The inclusion of general surgeons and gynaecologists alongside urologists is particularly relevant in low‐resource settings, where emergency urological care is frequently delivered outside specialist urology services.^13^, ^14^ Variation in training grade, specialty mix and faculty involvement across sites was intentional and reflected local workforce structures and educational priorities (Table 3).

Whether simulation‐based training can be adopted in low‐resource settings depends on practical factors such as who delivers emergency care, the availability of local teaching faculty, access to basic training infrastructure and how well programmes fit within existing institutions.^15^ The EUST model aligns with these constraints through its low‐resource training design, inclusion of clinicians from multiple surgical specialties and a staged approach to developing local faculty, and the consistent educational gains observed across different sites suggest that the model can be adapted to a range of training contexts.

Although this evaluation did not directly measure progression to faculty roles, the consistent replication of objective knowledge gains across heterogeneous cohorts provides indirect support for the feasibility of a staged ‘see one, do one, mentor one’ dissemination model.

The ‘see one, run one’ approach for centres without a strong Urolink connection has helped establish an additional motivated group of EUST trainers across a number of countries in sub‐Saharan Africa. Utilisation of senior trainees as faculty helped tremendously, as they form an important pool of educated SIM trainers to disseminate the EUST approach across differing parts of sub‐Saharan Africa as they relocate at completion of their training. They will provide a longitudinally sustainable, self‐supporting SIM training cohort for the future, which will undoubtedly facilitate the distribution of learning.

The faculty benefitted in three areas (a) pedagogical growth: faculty members benefited from a structured teaching environment, refining their ability to deliver SBE; (b) leadership development: the model allowed senior trainees to transition into junior faculty roles, enhancing their leadership and communication skills within the surgical team; and (c) international networking: faculty gained opportunities for cross‐border collaboration between Urolink (BAUS), the Urology Society of Ethiopia, other regional surgical colleagues (Uganda & Zimbabwe), and builds on the established relationship with The College of Surgeons of East, Central and Southern Africa (COSECSA).

### 4.2 Is EUST a model that could be disseminated into other low‐resource environments?

EUST is not a finished product but its transportability across borders suggests that with appropriate support it could be disseminated globally where educative opportunities are limited.^16^ It is achievable utilising support from a motivated and experienced SIM team co‐ordinating things for trusted partners. They can support and mentor local faculty both on‐site or remotely to ensure educational goals are achieved. EUST experience can also be shared by faculty observers new to SIM training, who can transition into organisers and mentors very rapidly using a ‘baton‐passing’ paradigm of ‘See one, run one, mentor one’.

There are some residual difficulties with this model, but these are predominantly technological, rather than technical, financial or related to Human Resources. There were some problems with delegates and faculty gaining online access to resource packs, which appeared to be as a consequence of internet accessibility and download speed when downloading large files, for which a technical solution is being sought. It also became obvious that total commitment to the project by the local organiser was a key requirement of the project when there was not an established relationship between Urolink and a LMIC Urology centre.

### 4.3 Cost‐effectiveness

By utilising locally sourced animal tissue (cow and goat) rather than expensive synthetic manikins, the course reduced simulation costs by thousands of dollars per session. Using digital theoretical modules prior to the course maximised the value of on‐site time, reducing the need for extended (and expensive) multiday hotel accommodation for faculty and delegates. The donation of 50 specialised surgical instrument sets by the Medi Tech Trust provided long‐term value, allowing sites to repeat the training without further equipment costs.

### 4.4 Sustainability

This is the core of the programme's sustainability. Local surgeons are trained to lead future courses, gradually reducing the need for international visiting faculty. Local Resource and Faculty Integration: Teaching faculty how to source and prepare tissue from local markets ensures that training can continue using readily available, low‐cost materials. We also encouraged a pre‐course visit by the local lead to a course there by prompting collaborations between different institutions and countries. Institutional Adoption: By partnering with a local educational lead and hospital leadership at Mengo, HUCSH and Kamuzu Central, the EUST is being considered for integration into the permanent fabric of regional surgical residency programmes.

In summary, EUST is a novel, affordable surgical simulation training tool, with objective, demonstrable, educational benefits, which will play a small, but important, part in the development of global benefit to urological patient's care in LMICs. Taken together, these findings suggest that EUST is a scalable and adaptable educational intervention capable of delivering meaningful knowledge gains across varied training contexts. Further work is required to evaluate longer term outcomes, including skill retention and the effectiveness of progression from participant to faculty roles.

## AUTHOR CONTRIBUTIONS

*Conceptualisation*: William James Gladstone Finch, Tilaneh Leyeh Demilow, Ramzi Yesuf, Chales Mabedi, Getaneh Tesfaye Teferi, Stephen R. Payne and Chandra Shekhar Biyani. *Methodology*: William James Gladstone Finch, Tilaneh Leyeh Demilow, Ramzi Yesuf, Getaneh Tesfaye Teferi, Stephen R. Payne and Chandra Shekhar Biyani. *Software*: NA. *Validation*: Tilaneh Leyeh Demilow, Ramzi Yesuf, Getaneh Tesfaye Teferi, Stephen R. Payne, Chandra Shekhar Biyani, William James Gladstone Finch, Fitsum Gebreegziabher Gebrehiwot, Vincent Medeyi and Chales Mabedi. *Formal analysis*: Folk‐Man Wong. *Investigation*: Tilaneh Leyeh Demilow, Ramzi Yesuf, Getaneh Tesfaye Teferi, Stephen R. Payne, Chandra Shekhar Biyani, William James Gladstone Finch, Fitsum Gebreegziabher Gebrehiwot, Linda Kayange, Matthew Trail, Vincent Medeyi and Chales Mabedi. *Resources*: Tilaneh Leyeh Demilow, Ramzi Yesuf, Getaneh Tesfaye Teferi, Chales Mabedi, Vincent Medeyi, Stephen R. Payne, Chandra Shekhar Biyani, William James Gladstone Finch and Fitsum Gebreegziabher Gebrehiwot. *Data curation*: Folk‐Man Wong, Tilaneh Leyeh Demilow, Ramzi Yesuf, Getaneh Tesfaye Teferi, Stephen R Payne, Chandra Shekhar Biyani and William James Gladstone Finch. *Writing—original draft*: William James Gladstone Finch, Tilaneh Leyeh Demilow, Ramzi Yesuf, Getaneh Tesfaye Teferi, Chales Mabedi, Folk‐Man Wong, Stephen R. Payne and Chandra Shekhar Biyani, *Writing—review and editing*: Tilaneh Leyeh Demilow, Ramzi Yesuf, Getaneh Tesfaye Teferi, Stephen R. Payne, Chandra Shekhar Biyani, Folk‐Man Wong, William James Gladstone Finch, Fitsum Gebreegziabher Gebrehiwot, Vincent Medeyi and Chales Mabedi. *Supervision*: Stephen R. Payne, Chandra Shekhar Biyani and William James Gladstone Finch. *Project administration*: Chales Mabedi, Vincent Medeyi, Getaneh Tesfaye Teferi, Ramzi Yesuf, Stephen R. Payne, Chandra Shekhar Biyani and William James Gladstone Finch.

## CONFLICTS OF INTEREST STATEMENT

WJGF and MT received a travel grant from Urolink; CSB and SP received a travel grant from BJUI. For the rest of the authors, no conflicts exist.
